# Genome Sequences of Three African Swine Fever Viruses of Genotypes IV and XX from Zaire and South Africa, Isolated from a Domestic Pig (Sus scrofa domesticus), a Warthog (Phacochoerus africanus), and a European Wild Boar (Sus scrofa)

**DOI:** 10.1128/MRA.00341-20

**Published:** 2020-08-06

**Authors:** S. Ndlovu, A.-L. Williamson, L. Heath, O. Carulei

**Affiliations:** a Division of Medical Virology, Department of Pathology, Faculty of Health Sciences, University of Cape Town, Cape Town, South Africa; b Agricultural Research Council—Onderstepoort Veterinary Research, Pretoria, South Africa; c Institute of Infectious Diseases and Molecular Medicine, Faculty of Health Sciences, University of Cape Town, Cape Town, South Africa; DOE Joint Genome Institute

## Abstract

We report here the genome sequences of three African swine fever virus isolates obtained from a domestic pig (Zaire [Zaire]), a warthog (RSA/W1/1999 [South Africa]), and a European wild boar (RSA/2/2004 [South Africa]) belonging to genotypes IV, XX, and XX, respectively. This report increases the number of genotype XX, wild boar, and warthog reference sequences available.

## ANNOUNCEMENT

African swine fever virus (ASFV) is the only species belonging to the *Asfivirus* genus of the *Asfarviridae* family and is comprised of a complex, linear, double-stranded DNA genome characterized by identical, terminal, inverted repeats and hairpin loops at both ends. The length of the genome is between 170 and 190 kbp and encodes 150 to 167 open reading frames (ORFs) (1–4). ASFV causes African swine fever, a hemorrhagic disease affecting domestic pigs and wild boars that results in mortality rates approaching 100%. Currently, there is only one genome sequence (GenBank accession number AY261366) of an ASFV isolate from a warthog and one genome sequence (GenBank accession number AY261363) of a genotype XX isolate. The lack of described and fully sequenced isolates from different geographical regions and hosts, including warthogs, ticks, wild boars, and domestic pigs, is one of the major gaps identified in the ASFV field (5–7), and thus we report on three novel ASFV isolated from different hosts.

The ASF viruses were originally isolated on primary murine bone marrow macrophages (PBMC) from tissue samples collected from infected suids. The first virus, Zaire, was isolated from a domestic pig in Zaire in 1977 and subsequently passaged on rhesus monkey kidney epithelial cells (LLC-MK2) and African green monkey cells (Vero). RSA/W1/1999 and RSA/2/2004 were isolated from a warthog from South Africa in 1999 and a European wild boar from South Africa in 2004, respectively. The viruses were revived from frozen stock by a single passage on freshly prepared PBMC (RSA/W1/1999 and RSA/2/2004) or Vero cells (Zaire). Viral DNA was extracted from the cell suspensions using the High Pure PCR template preparation kit (Roche Diagnostics, Germany). The NEBNext microbiome DNA enrichment kit (New England Biolabs, USA) was used to deplete host methylated DNA. The isolated DNA was amplified with the GenomiPhi v2 DNA amplification kit (GE Healthcare, USA), and conventional PCR amplification and sequencing of the C-terminal *p72* gene region were used to confirm the ASFV nucleic acid presence and genotype, respectively, as described previously (8). CLC Genomics Workbench v9.0.1 was used to generate an unrooted maximum-likelihood tree (HKY model with rate and topology variation and 1,000 bootstrap replicates) based on the multiple sequence alignment (MSA) of partial (399-nucleotide [nt]) *p72* sequences from 43 ASFV isolates. Genome sequencing was performed on an Illumina MiSeq instrument using the Nextera XT DNA sample preparation kit and the v3 reagent kit for 2 × 300-bp paired-end reads (Illumina, USA). The tagmented DNA was mixed with index adapter following the manufacturer’s instructions; then, 15 μl of the Nextera PCR master mix (NPM) was added to each well, the mix was pipetted 10 times, and the plate was sealed. The plate was then centrifuged at 280 × *g* at 20°C for 1 min and placed on the thermal cycler, and the NXT PCR program was run. *De novo* assembly of all reads was performed using CLC to generate genomic contigs. Smaller nonspecific contigs were identified as belonging to the host genome. GATU (9) was used to annotate the genomes, using BA71V (10) as the reference; CLC was used to search for ORFs in regions that were missed by GATU, and a BLASTx search was performed against the NCBI nonredundant (nr) protein database to confirm the ORFs. All ORFs intact at their 5′ end but comprising less than 80% of ortholog length were annotated as truncated genes, while those ORFs disrupted at their 5′ end were annotated as fragmented genes (11).

The Zaire, RSA/W1/1999, and RSA/2/2004 isolates showed similar genome statistics in terms of genome length, number of ORFs, and G+C content (Table 1). The genomic termini were not sequenced.

**TABLE 1:** Sequencing and basic genome statistics of three novel ASFV genomes

	Data for isolate:		
Statistic	Zaire	RSA/W1/1999	RSA/2/2004
Total no. of reads	8,525,098	3,726,962	3,749,576
No. of virus-specific reads	4,277,735	2,236,520	2,156,036
Coverage (×)	6,924	3,621	3,431
Genome length (bp)	185,337	185,293	188,502
No. of ORFs	166	167	167
G+C content (%)	38.6	38.6	38.6
Genotype	XX	IV	XX
GenBank accession no.	MN630494	MN641876	MN641877
SRA accession no.	PRJNA587577	PRJNA587581	PRJNA587591

**FIG 1:**
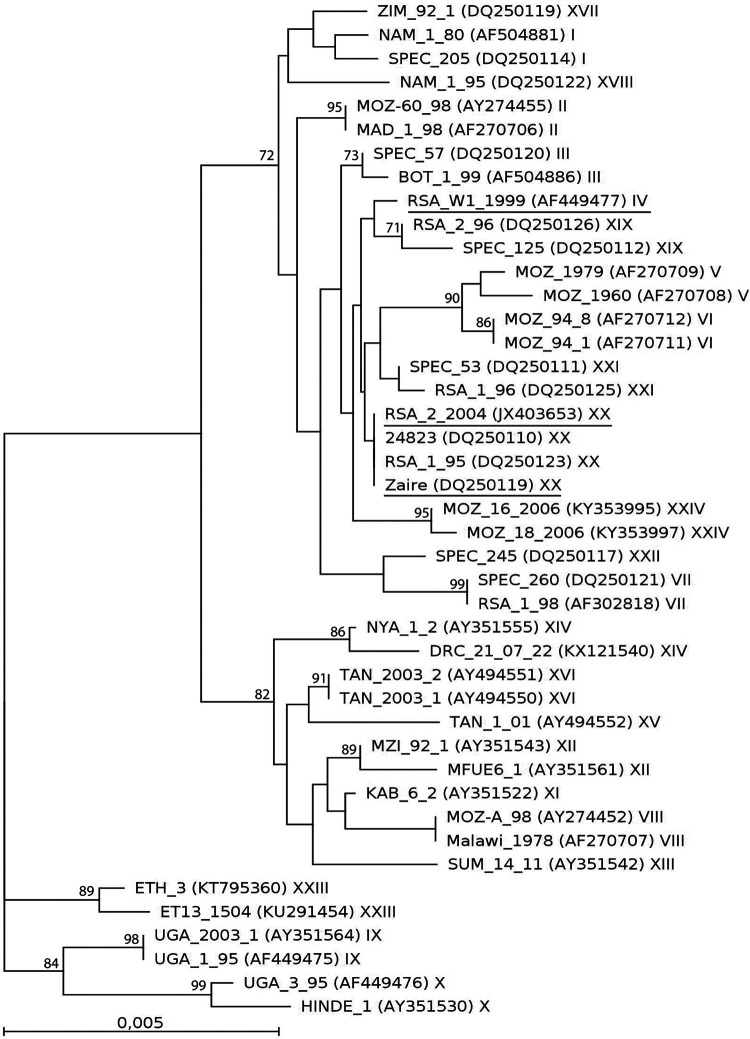
Unrooted maximum-likelihood phylogenetic tree constructed from the alignment of partial *p72* sequences (399 nt) from 43 ASFV isolates, including three novel sequences described in this study (bold text). Isolate names are followed by accession numbers and *p72* genotypes (I to XXIV). Bootstrap values of ≥70% are shown at branch nodes, and the scale bar represents nucleotide substitutions per site.

Multiple sequence alignment and phylogenetic analysis of the C terminus of the *p72* gene showed that Zaire belongs to genotype XX, RSA/W1/1999 to genotype IV, and RSA/2/2004 to genotype XX (Fig. 1), with Zaire showing the closest BLASTn (NCBI nr database) identity to Benin/97/1 from Benin (genotype I; GenBank accession number EF121428), RSA/W1/1999 to Lillie from South Africa (genotype XX; DQ250109), and RSA/2/2004 to wart from Namibia (genotype IV; AY578706). A BLASTn search of the full-genome sequences against the NCBI nr database with default parameters showed that the genome sequences most similar to Zaire, RSA/W1/1999, and RSA/2/2004 were Pretoriuskop/96/4 (AY261363; genotype XX; 97% identity and 99% coverage) and Warthog (AY261366; genotype IV; 98% identity and 99% coverage), respectively. These data add the second warthog genome sequence and two more genotype XX isolates from a domestic pig and a wild boar to the collection of sequences available for comparative genomic analysis.

### Data availability.

The ASFV genome sequences in this report are available in GenBank under accession numbers MN630494 (Zaire), MN641876 (RSA/W1/1999), and MN641877 (RSA/2/2004). The raw reads are available in the SRA under accession numbers PRJNA587577 (Zaire), PRJNA587581 (RSA/W1/1999), and PRJNA587591 (RSA/2/2004).
